# Work-related support in clinical care for patients with a chronic disease: development of an intervention

**DOI:** 10.1007/s10926-022-10032-z

**Published:** 2022-05-20

**Authors:** Maarten Butink, Desiree Dona, Annelies Boonen, Marlies Peters, Vera Baadjou, Theo Senden, Angelique de Rijk

**Affiliations:** 1 Department of Internal Medicine, Division of Rheumatology, Care and Public Health Research Institute (CAPHRI), Maastricht University Medical Centre+ (MUMC+), P. Debyelaan 25, 6229 HX Maastricht, The Netherlands; 2 Department of Social Medicine, Care and Public Health Research Institute (CAPHRI), Maastricht University, Duboisdomein 30, 6200 MD Maastricht, The Netherlands; 3 Department of Human Resources/Occupational Health Services, Radboud University Medical Centre, Geert Grooteplein Zuid 10, 6525 GA Nijmegen, The Netherlands; 4 Adelante Rehabilitation Centre, Maastricht, The Netherlands and Department of Rehabilitation Medicine, Care and Public Health Research Institute (CAPHRI), Maastricht University, Universiteitssingel 40, 6200 MD Maastricht, The Netherlands

## Abstract

**Background:**

Patients with a chronic disease are more vulnerable in the labor market, and work-related support in clinical care would enhance the timely support greatly needed in each phase of their working life. This paper describes the development of a generic stay-at-work intervention to provide work-related support in clinical care to patients with a chronic disease.

**Methods:**

Steps 1–4 of Intervention Mapping (IM) were combined with action research principles. A needs assessment (Step 1) involved the project group formation, a literature review, qualitative studies with healthcare professionals (HCPs; n = 9) and patients (n = 10), consultation with financial staff and testing, and resulted in objectives (Step 2). Guided by methods and applications (Step 3), the intervention was developed, tested and finalized (Step 4).

**Results:**

The needs assessment revealed the importance of behavioral change in HCPs, including changing attitude, self-efficacy, and social influence. For that purpose, a pathway and training sessions were developed. Testing these unveiled the need for practical tools and intervision. The final intervention comprises a care pathway as part of working routines, including screening, risk stratification, and tailored support. Practical tools, training sessions, and intervision for HCPs were developed.

**Conclusions:**

Combining IM with action research principles resulted in a generic stay-at-work intervention in clinical care via behavioral change in HCPs. A generic care pathway, practical tools, training sessions, and intervision were developed. More specific alignment to specific patient groups is possible. To implement the intervention in another hospital, the local context, (financial) resources, and the national legislation should be considered.

**Supplementary Information:**

The online version contains supplementary material available at 10.1007/s10926-022-10032-z.

## Introduction

Earlier diagnosis and more effective medical interventions have resulted in the improved survival of persons with chronic diseases and co-morbidity. Consequently, an increasing proportion of people in Western societies have one or more chronic diseases and are at risk of experiencing restrictions in functioning and participation [1–3]. At the same time, the double ageing forces societies with a social welfare system to adopt activating social policies, resulting in efforts to maximize the work participation of all persons, including those with one or more chronic diseases [4, 5]. However, the work participation rates of patients with chronic diseases in Europe is low, varying between 25 and 57% [6]. Once withdrawn from the labor force, return to paid employment is difficult, and even more so for persons with chronic diseases [7]. Nevertheless, individuals prefer in general to continue working, because of economic, social, and functional benefits [8].

Several clinical and workplace interventions have been developed and evaluated for patients with a chronic disease that aim at improving work participation (e.g. [9–14]). However, few of them focus on retaining work participation within the setting of clinical care, and most interventions provide one-time support to patients without follow-up and adjustment of support (e.g., [11–14]). Moreover, the growing group of self-employed people, ranging in Europe from 8.5 to 31.9% in 2020 in Denmark and Greece, respectively, and 17.2% in the Netherlands [15], are more likely to rely on self-funding of healthcare and work-related support in many countries, as the social benefits for this group are still limited. This makes this ‘new’ group of self-employed in often lower-income professions at potential risk for adverse health and work outcomes when they are diagnosed with a chronic disease. This group has received limited attention in current work participation studies [16]. It is therefore important to include these people in new interventions aimed to support work participation.

As persons with a chronic disease are commonly under the care of healthcare professionals (HCP), there is an opportunity within the healthcare system to provide work-related support throughout the journey [17–20]. We focused on those who still had employment, as return to work (RTW) in those who lost their employment is difficult, not in the least because often (suitable) jobs on the labor market are lacking [21]. Paying continuous systematic attention to stay-at-work and providing tailored support should become part of these professionals’ routine and clinical reasoning [22]. This is also increasingly recommended by national socio-economic or medical advisory reports and the scientific literature [17, 20, 23–25]. Staying at work is considered as working without sick leave or only short periods of sick leave, in line with e.g. Van Hees et al. [26].

A survey in the Netherlands among 104 rheumatologists and 103 rheumatology nurses (response rate 40% and 50%, respectively) confirms that work participation is a relevant treatment goal for HCPs. However, the respondents also emphasized the need for accurate tools to identify patients at risk and work-related risk profiles, and get insight into cost-effective support for patients at risk [27].

Clearly, there is a need to develop a generic stay-at-work intervention to provide work-related support in clinical care for patients of several chronic diseases even before they may be hindered by their disease from performing their work. The chain of routinely discussing work participation to providing work-related support when indicated will require a behavioral change of HCPs. The Intervention Mapping (IM) approach is a resourceful tool to develop an intervention that is highly dependent on behavioral change [12, 28, 29]. IM is a step-wise, systematic approach for intervention development to stimulate researchers to oscillate between steps and to connect evidence-based research to practice [30, 31]. To understand practical needs and facilitate integration in clinical care, we combined IM with action research [32]. More specifically, we aimed to test draft parts of the generic intervention during the intervention development at the rheumatology outpatient clinic at a Dutch university hospital, and subsequently apply to two other patient groups. This paper describes the development of a generic stay-at-work intervention to provide work-related support to patients with a chronic disease in clinical care.

## Methods

### Design

The first four steps of IM (1. Needs assessment, 2. Objectives, 3. Methods and Practical Applications, and 4. Intervention Production) were applied to develop the intervention [31]. Step 5 (Adoption and Implementation) and 6 (Evaluation Planning) will be addressed in planned research. The project group proposed a combination of IM and action research in all steps, which was seen as an opportunity to include the experiences of those involved in the clinical care setting [32, 33]. Parts of the draft intervention were tested by medical specialists and nurse specialists (HCPs) from the participating outpatient clinic. Experiences collected from these HCPs and their patients were added to the incremental development process to better integrate the intervention into clinical care.

The steps were coordinated by a project group with expertise in a broad group of chronic diseases, including a health scientist with a master’s degree in Work, Health and Career (MB), a medical specialist in a university hospital and rheumatologist with a research interest in work participation in rheumatic diseases (AB), a professor in work and health specializing in re-integration into work with experience with IM and work-related support in clinical settings (AdR), a medical specialist in rehabilitation medicine (VB), a project and research coordinator in clinical occupational care (MP), and two clinical occupational physicians (DD, TS) with experience in work-related support in a second university hospital. Members of the project group brought in the occupational physician perspective [34]. Many (online) meetings were organized to discuss the IM steps. The aim of the consecutive steps [31] and the activities undertaken by the project group are summarized in Table 1. For a comprehensive explanation of IM, consult Bartholomew et al. [31].

**Table 1:** Aim of the steps and activities undertaken by the project group

	*Aim*	*Activities*
*Step 1*	To explore needs that should be addressed in the intervention.	Different sources were used as a broad inventory of needs, views, resources, experiences, and opportunities, and formed a starting point for the intervention development. Sources are the project group’s expertise, qualitative study with HCPs, qualitative study with patients, systematic literature review, consultation with financial staff member, and testing parts of the intervention at one outpatient clinic.
*Step 2*	To state the overall intervention goal, supplemented by behavioral outcomes, performance objectives, and change objectives.	The assessed needs (Step 1) were discussed within the project group, and a logical model of change was chosen to change behavior. An overall intervention goal, including behavioral outcomes, performance objectives, and change objectives, was chosen.
*Step 3*	To choose change methods, strategies, and practical applications to deliver the change objectives.	To deliver the change objectives (Step 2), the project group gathered several evidence-based change methods, strategies, and practical applications.
*Step 4*	To produce the intervention and materials.	Based on the practical applications (Step 3), the project group developed the intervention and materials. Parts of the intervention and its materials were tested in clinical care and resulted in adjustments to the intervention and materials.

## (Step 1) What needs to be addressed in the intervention?

The project group met regularly to share expertise on work-related support. Meetings were organized with the project group to improve the intervention’s sustainability in the local context based on experiences in other contexts. Meeting documents were used as input. Several other sources were consulted to complete the inventory of needs. A qualitative study to collect HCPs’ views (n = 9) on providing work-related support in clinical care (such as the barriers, needs, and patients’ perspective) was conducted (unpublished thesis, 2020). Patients with rheumatoid arthritis (n = 10) were interviewed while testing the intervention, to collect views on work-related support in clinical care (unpublished thesis, 2021). A systematic literature review on interventions to identify effective work-related support to patients with rheumatic and musculoskeletal disorders and a non-systematic review of prediction models for long-term absence were performed (unpublished review in preparation). The hospital’s financial staff member was asked for advice and frameworks in which the work-related support should be embedded financially. Following principles of action research [32], experiences gained while testing parts of the intervention at one of the outpatient clinics (rheumatology) delivered additional needs.

All assessed needs were summarized by the project group. Terminology used from the needs assessment onwards is presented in the glossary (Online resource 1).

## (Step 2) What are the intervention objectives?

The overall intervention goal was supplemented with behavioral outcomes *(what is intended to change?)*, performance objectives *(what actions are needed to perform the desired behavior?)*, determinants *(what underlying determinants contribute to the behavior?)*, and change objectives *(what needs to change in the determinants of the behavior to accomplish the performance objective?)*, and resulted in a matrix [31].

## (Step 3) What methods, strategies, and practical applications are needed for the intervention?

The project group gathered several evidence-based methods, strategies, and practical applications to target the change objectives. The proposed ideas for practical applications were discussed with HCPs as part of the action research [32] and were included in the matrix.

## (Step 4) What does the intervention and its materials look like?

The project group transformed the selected practical applications into the intervention, including materials. All materials were tested by HCPs at the outpatient clinic (action research), and their input was added to the incremental development. Project group members (MB, AdR) and a graphic designer developed the materials.

## Results

### (Step 1) What needs to be addressed in the intervention?

### Setting

The intervention users were medical specialists and nurse specialists (HCPs) active in a Dutch university hospital. The HCPs applied the intervention during their usual working routine to their working patients with a chronic disease.

### Needs assessment

Multiple needs were derived from the sources and are summarized in Table 2. The need for a work-related support care pathway alongside the patient’s medical journey, knowledge and skills, tools, and feeling supported appeared important for HCPs. Patients expressed the need to discuss work (problems) during consultations with their HCP, and to receive support tailored to their individual situation when indicated.

**Table 2:** Overview of needs by sources, resulting in summarized needs for the intervention development

HCPs’ needs	Sources						Summarized needs
	**Project group**	**Qualitative study HCPs**	**Qualitative study patients**	**Systematic literature review**	**Financial staff member**	**Testing intervention (action research)**	
Need for a generic work-related support intervention, depicted in a care pathway	X						A. Need for a care pathway
Need to integrate work-related support with hospital’s existing financial flows	X				X		
Need to be able to discuss work participation in regular consultations and stratify patients according to their level of problems	X	X	X	X			B. Need for knowledge and skills
Need for designated HCPs to conduct a problem assessment	X	X		X			
Need for designated HCPs to provide tailored work-related support	X		X	X			
Need to follow-up on their patients regarding work participation and work-related support	X						
Need to have knowledge on work-related support, social legislation, problem assessment, and providing tailored work-related support	X	X				X	
Need to have a positive attitude towards providing work-related support	X						
Need to learn about work-related support in interactive training sessions		X				X	
Need to tailor work-related support to individual patients	X		X	X		X	
Need for practical tools to facilitate work-related support in scarce consultation time		X				X	C. Need for tools
Need to feel supported by colleagues in providing work-related support		X				X	D. Need to feel supported

*HCP = healthcare professional*

## (Step 2) What are the intervention objectives?

The project group decided on the overall intervention objective being ‘Providing work-related support in clinical care to promote sustainable and healthy work participation of patients with a chronic disease that have paid work,’ including behavioral outcomes, performance objectives, determinants, and underlying change objectives. For each behavioral outcome, the accompanying performance objectives, underlying determinants, and associated change objectives are presented in the matrix (Table 3, Step 2). The behavioral outcomes were linked to the summarized needs of Step 1 (Table 3, Step 1).

**Table 3:** Matrix of Steps 1–4 of the intervention development

Step 1	Step 2			Step 3			Step 4
**Summarized needs**	**Intervention objectives**			**Methods, strategies, and practical applications**			**Intervention and its materials**
	**Behavioral outcome 1: HCPs are able to conduct a work-related screening**						
	*Performance objective (PO)*	*Determinant*	*Change objective (CO)*	*Method*	*Strategy*	*Practical application*	
A. Need for a care pathway B. Need for knowledge and skills C. Need for tools	PO 1.1 HCPs are aware of the importance of screening patients at risk for work participation problems in early stages to provide work-related support to a subgroup of patients	Attitude	A 1.1 HCPs express positive feelings about their responsibility to identify patients at risk for work-related support	• Continuous medical education • Practical tools • Clinical reasoning	• Outreach programs • Workshops • Academic detailing • Practical tools • Clinical reasoning	• Educational formats to target HCP’s attitude and self-efficacy • Development of a care pathway to increase self-efficacy	Training session 1, intervision
	PO 1.2 HCPs are able to ask about work participation (and restrictions) during regular consultations	Self-efficacy	SE 1.2 HCPs express confidence in their ability to ask about work participation (and restrictions) during regular consultations				Care pathway, screening card, training session 1, intervision
	PO 1.3 HCPs are able to identify patients at risk for prolonged sick leave or withdrawal from work	Self-efficacy	SE 1.3 HCPs express confidence in their ability to screen patients for work participation problems				Care pathway, screening card, training session 1, intervision
	**Behavioral outcome 2: HCPs are able to stratify patients for tailored support**						
A. Need for a care pathway B. Need for knowledge and skills C. Need for tools	PO 2.1 HCPs are able to stratify patients using clinical reasoning and practical tools	Self-efficacy	SE 2.1 HCPs express confidence in the accuracy of stratification approaches and in their ability to apply such stratification tools	• Continuous medical education • Practical tools • Clinical reasoning	• Outreach programs • Workshops • Academic detailing • Practical tools • Clinical reasoning	• Development of a care pathway to increase self-efficacy • Educational formats to target HCP’s self-efficacy • Practical tools to improve HCP’s self-efficacy	Care pathway, screening card with stratification, training session 1
	PO 2.2 HCPs are able to refer patients to designated HCPs for tailored support	Self-efficacy	SE 2.2 HCPs express confidence in their ability to refer patients to designated HCPs for additional tailored work-related support				Care pathway, screening card with stratification, training session 1
	**Behavioral outcome 3: Designated HCPs are able to provide tailored support**						
A. Need for a care pathway B. Need for knowledge and skills C. Need for tools D. Need to feel supported	PO 3.1 HCPs providing work-related support for patients with low-complexity problems are able to correctly understand the nature of the problem, using clinical reasoning	Self-efficacy	SE 3.1 HCPs providing tailored support to patients with low-complexity problems express confidence in their ability to conduct a basic problem assessment, using clinical reasoning	• Continuous medical education • Practical tools • Clinical reasoning • Local opinion leaders	• Outreach programs • Practical tools • Clinical reasoning • Discuss facilitation of integration into financial flows	• Development of a care pathway to increase self-efficacy • Educational formats to target HCP’s attitude and self-efficacy • Practical tools to improve HCP’s attitude and self-efficacy • Appoint opinion leaders to target HCP’s social influence • Alignment of the care pathway with financial flows	Care pathway, conversation cards, training session 2, intervision
	PO 3.2 HCPs providing work-related support for patients with high-complexity problems are able to correctly understand the nature of the problem in relation to the diverse medical problems, using clinical reasoning	Self-efficacy	SE 3.2 HCPs offering HCPs providing tailored support to patients with high-complexity problems express confidence in the ability to assess their problems in relation to the diverse medical problems, using clinical reasoning				Care pathway, conversation cards, MOWS, flyer, training session 2, intervision
	PO 3.3 Designated HCPs are able to prioritize work-related problems	Self-efficacy	SE 3.3 Designated HCPs express confidence in their ability to prioritize the problems				Care pathway, conversation cards, training session 2, intervision
	PO 3.4 Designated HCPs are aware of publicly available interventions, advice, and recommendations to support patients	Attitude	A 3.4 Designated HCPs express positive feelings about the benefits of publicly available interventions, advice, and recommendations to support patients				Care pathway, MOWS, training session 2, intervision
	PO 3.5 Designated HCPs are able to provide tailored support	Self-efficacy	SE 3.5 Designated HCPs express confidence in their ability to provide tailored support				Care pathway, conversation cards, training session 2, intervision
	PO 3.6 HCPs feel facilitated by means of time and finance	Social influence	SI 3.6 HCPs recognize the facilitation by means of time and finance				Care pathway (including alignment with financial flows)
	PO 3.7 HCPs feel supported by their colleagues, working environment, and experts to provide work-related support	Social influence	SI 3.7 HCPs recognize the support by their colleagues, working environment, and experts to provide work-related support				Care pathway (including opinion leaders)
	**Behavioral outcome 4: HCPs are able to follow-up on their patients**						
B. Need for knowledge and skills	PO 4.1 HCPs are aware of the importance of following up their patients in work-related support	Attitude	A 4.1 HCPs express positive feelings about the benefits of following up their patients in work-related support	• Continuous medical education	• Outreach programs	• Development of a care pathway to target HCP’s attitude and self-efficacy • Educational formats to target HCP’s attitude and self-efficacy • Practical tools to improve HCP’s attitude and self-efficacy	Care pathway, training sessions 1 and 2, intervision
	PO 4.2 HCPs are able to follow up these patients	Self-efficacy	SE 4.2 HCPs express confidence in their ability to follow up these patients				Care pathway, screening card, conversation cards, MOWS, flyer, training sessions 1 and 2, intervision

*HCP = healthcare professional, A = attitude, SE = self-efficacy, SI = social influence, MOWS = Map with Options for Work-related Support*

The project group expected that behavioral change of HCPs would be the key element of the intervention to provide work-related support. To address this, a logic model of change was applied. The ASE-model was chosen [35]. Following this model, behavioral change is determined by three core determinants: Attitude, social influence, and self-efficacy. First, *attitude* consists of beliefs and thoughts about the capability to adopt the behavior [35, 36]. Second, *social influence* consists of the perception of others carrying out the behavior or norms that others have regarding this behavior [35, 36]. Last, *self-efficacy* refers to a person’s perception of their capability to adopt the required behavior [35]. These determinants were translated into change objectives for the work-related support intervention (Table 3, Step 2).

## (Step 3) What methods, strategies, and practical applications are needed for the intervention?

Focusing on the change objectives, available evidence-based methods, strategies, and practical applications were collected in Step 3 and presented in Table 3, Step 3.

Based on Mostofian et al. [37], there is evidence that *continuous medical education* and *local opinion leaders* are effective methods for behavioral change among medical specialists and likely by extension also for other HCPs, such as nurses. Continuous medical education refers to information transfer via training sessions or meetings, and entails active forms [38]. The strategies comprise organizing academic detailing (one-on-one meeting with HCP to change the behavior), outreach programs (academic detailing in group meetings), or workshops (more interactive forms that enable HCPs to practice materials). Continuous medical education will change attitudes and increase self-efficacy (Table 3, Step 3). The term ‘Local opinion leaders’ refers to influential colleagues who can change their colleagues’ behavior [38]. Appointing influential people as local opinion leaders is considered the strategy and practical application to change social influence. As a third method, *practical tools* are needed to improve the self-efficacy of HCPs (Tables 3, Step 3). These tools can provide practical guidance in the process of screening, stratification, problem assessment, and providing tailored work-related support. To remain close to the professionalism of the HCPs, *clinical reasoning* is regarded the preferred method to translate patient’s work-related problems into support. In clinical reasoning, the purposeful use, combination, weighing, and application of knowledge, cognitions, reflections, clinical experience, best practices, and observations by HCPs can achieve patient-centered, work-related support that may also deviate from protocols or guidelines where necessary [39].

In this step, the action research yielded no additional input.

## (Step 4) What does the intervention and its materials look like?

**Fig. 1:**
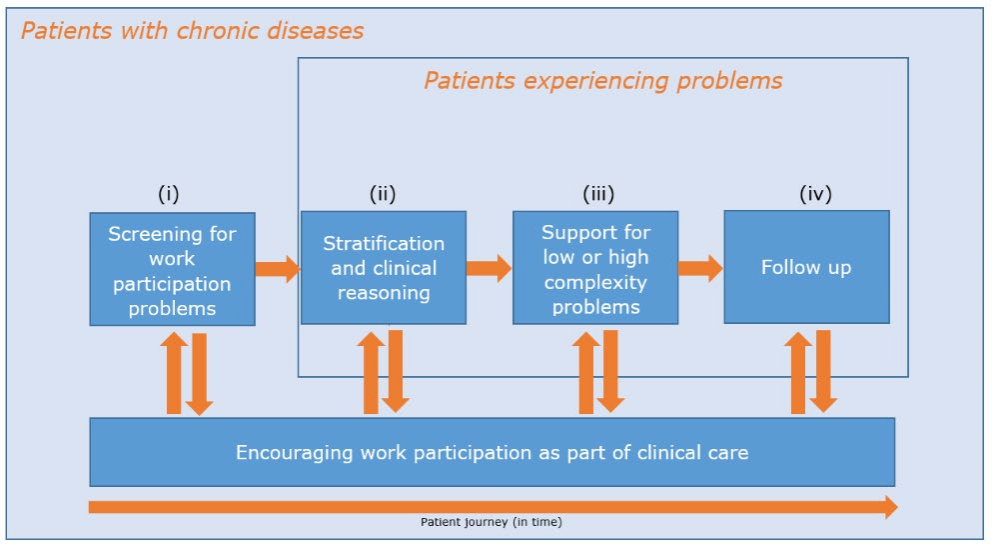
Care pathway of work-related support in clinical care

The care pathway comprises: (i) screening those at risk for long-term sickness absence, (ii) in-depth problem identification and stratification, (iii) tailored work-related support towards healthy and sustainable participation in paid work, and (iv) follow-up (Fig. 1). In line with patients’ needs, HCPs incorporate this in a process of clinical reasoning around work participation during each regular care visit of the patient (Tables 2 and 3, Step 4). To screen patients at risk for long-term sickness absence or withdrawal from work, presenteeism (reduced work ability or productivity while at work) and/or recent or current absenteeism have been identified as strong generic predictors, and are thus sensitive means to identify which persons might benefit from work-related support [40]. For persons at risk, issues should be determined and analysed in order to arrive at a stratification for risk for long-term sickness absence or withdrawal from work, and find potential support options. In this process, the HCP combines the components of the International Classification of Functioning, Disability, and Health (ICF), representing the biopsychosocial model of health [41], along with tacit knowledge and personal experiences with the patient. In this process, the identification of facilitators is equally important as the identification of barriers. While clinical reasoning might seem a burdensome task, with accumulating experience HCPs will recognize patterns and profiles that facilitate the stratification and agreement of shared support options. Notwithstanding, problems can be clearly too complex for the regular HCP, and advice is from or referral to an designated HCP trained in work and health (preferably working in the same hospital or care setting) is indicated. This can be shaped by a central place in the hospital for all types of chronic diseases and conducted by a designated HCP, for example, a medical specialist in rehabilitation medicine or a clinical occupational physician.

Regularly updating knowledge on the effectiveness of work interventions or new insights into risk profiles or regulations in the social security system must be integrated into the risk classification and care plan. Examples of low-complexity and high-complexity problems with potential support options are presented in Table 4.

**Table 4:** Example cases and support options in work-related support

Case example of low-complexity problem	Case example of high-complexity problem
Mrs. L is a 45-year-old woman with rheumatoid arthritis. Despite adequate anti-inflammatory treatment and adequate disease control, she experiences symptoms of fatigue leading to a lower quality of life and prevents her from taking care of the children (ages 7 and 10) in the morning. In addition, this also particularly hinders her work as a secretary (32 h per week) in the morning. She questions what she can do to give this fatigue a place and to continue doing her work well.	Mrs. H is a 45-year-old woman with rheumatoid arthritis and other co-morbidities. Despite adequate treatments and adequate disease control, she has an unhealthy lifestyle, low coping strategy, and exhibits depressive symptoms. In the morning, she experiences morning stiffness, which interferes with caring for her children (ages 7 and 10) and performing her job as a secretary (32 h per week). Coming home from work, she is exhausted and has no energy to socialize with family and friends and do sports. She has difficulty living with the diagnoses and finds working in conjunction with her diseases burdensome.
**Possible support options by designated HCP**	**Possible support options by designated HCP**
• Advise Mrs. L to get support in the morning in taking care of her children (such as discussing support from her partner) • Advise Mrs. L to discuss this practical issue with her employer/manager. The HCP can support her to start the conversation and provide options for support (such as adjusting working hours or partially working from home)	• Advise Mrs. H to get support in the morning in taking care of her children (such as discussing support from her partner) • Coordinate support to improve (in a combined manner or after prioritization; in collaboration with a physiotherapist, social worker and/or psychologist): o her lifestyle o her coping strategies o her depressive symptoms • Coordinate and advise Mrs. H to consult her occupational physician for a preventive consult and/or discuss work accommodations (such as adjusting start time or having more flexible working hours) with her employer/manager

In a setting where prevention is a staple factor, it is important to briefly point to the importance of work participation in relation to the meaning of life, social inclusion, and economic independence, including for persons who were not at risk for adverse work outcomes, and encouraging them to continue work in a healthy way [42].

The intervention was tested at the outpatient clinic by rheumatologists and rheumatology nurses to incrementally develop the intervention. These HCPs were engaged to perform screening and stratification and formulate care plans. To accommodate for stronger time constraints in the visit with the medical specialist, the specialists were allowed to refer patients who desired support about sustainable work or experience restrictions in work ability to the rheumatology nurse specialist to elaborate the problems of low complexity. A medical specialist in rehabilitation medicine and specialized in work participation organized a ‘work participation clinic’ for problems of higher complexity. Both the medical specialist in rehabilitation medicine and the rheumatology nurse specialist were the ‘designated HCP’ who provides support for patients with either low- or high-complexity problems.

The pathway of work-related support, including the support by HCPs, was tested at the outpatient clinic. For this, the pathway was aligned with current resources and largely to financial flows. During testing the intervention and pathway, HCPs still indicated the limited time to pay attention to the work participation of their patients during the testing of the intervention. Apparently, HCPs give priority to other tasks, such as reducing disease activity, improving physical function or screening for comorbidities (unpublished thesis, 2020). Moreover, during testing and interviews with HCPs and patients, it became clear that there is still insufficient awareness about the possibility of discussing work participation during consultations (unpublished thesis, 2021). The practical tools that can support this were therefore considered important for further intervention development.

## Practical tools

To support the behavioral change of the HCPs, practical tools were developed and tested at the outpatient clinic. First, a screening card was designed to facilitate HCPs in screening work participation problems and deciding whether referral would be required. The front side of the card contains two questions to help HCPs in identifying persons at risk for long-term absence or work disability. The back side provides suggestions for referring the patient to a designated HCP, depending on the complexity of the problem. Patients without problems do not require further support at that time and are encouraged to continue their work.

Second, five conversation cards for the designated HCPs were developed for in-depth exploration and development of a care plan of patients with either simple or complex problems. The first four cards contain trigger questions to use during the problem assessment, based on four domains: health, work, support, and personal characteristics (based on [41]). The fifth card stimulates the HCP to jointly prioritize the most urgent problem that should be targeted and discuss possible options for work-related support. This latter card acts as a bridge to the Map with Options for Work-related Support (MOWS). The MOWS summarizes accessible options for work-related support per domain in the problem assessment, such as advice to communicate with employer or occupational physician on e.g., work accommodations [43, 44], job coaching [45], referral to employee insurance agency or financial advisors.

Finally, and based on input from HCPs and patients while testing the intervention, a separate flyer for patients with relevant websites regarding the Dutch social legislation, patient associations, and advice was developed, to answer the limited time in consultations (unpublished thesis, 2021). Only minor suggestions appeared, when HCPs tested all materials in practice. (All materials, which are in Dutch, can be requested from the corresponding author.)

## Training sessions and intervision

Two training sessions were developed to train HCPs to provide work-related support in their clinical practice and educate them in the use of the practical tools. All training participants received training materials beforehand to prepare the online training sessions. In addition, intervision was organized to recall what was learned and gain new knowledge from colleagues and experts. For each intervision session, an expert is invited, such as an insurance physician and an occupational physician, to share specific knowledge on work disabilities, (partial) disability insurance, legislation, work accommodations and to explain their roles and responsibilities in relation to work-related problems [34]. The project group will organize intervision multiple times upon request from HCPs. The intervision focuses on one or more topics brought in by HCPs. Patient cases that are challenging regarding work-related issues are introduced, the problem is collaboratively defined and solutions are defined with colleagues [38]. An overview of the format and content of the training sessions and intervision is presented in Online Resource 2.

## Discussion

This paper describes the development of a generic intervention to provide work-related support to patients with a chronic disease as part of routine clinical care. The focus of this intervention was to change the behavior of medical specialists and nurse specialists to provide work-related support. To develop a generic stay-at-work intervention in a setting with little experience with the topic of work participation, we combined Intervention Mapping (IM) with the principles of action research [31, 32]. Intervention parts were tested at one of the participating outpatient clinics and gave an initial impetus for the incremental development of the intervention for other patient groups. The need for knowledge, skills, and practical tools among HCPs appeared important. To change the behavior of HCPs, a generic care pathway, practical tools, training sessions, and intervision were developed.

Work participation has been a focus of multiple interventions but with limitations: they were predominantly focused on return to work or did not include the role of HCPs in work-related support to prevent work participation problems. In the published literature on non-pharmacological interventions to stay at work, accurate screening of patients at risk to provide support received little attention, and a focus to integrate work-related components to clinical care appeared to be limited [11–14]. This study adds to the literature an intervention development process to provide work-related support in clinical care, via the behavioral change of HCPs.

The current work-related support intervention differs from other interventions that promote work participation. First, previous interventions focused on a specific target group, for example patients with a cancer diagnosis [12, 14], while this intervention focuses on stay-at-work of (self-)employed patients with any chronic disease. In addition, other interventions were designed to provide short-term support without a change in clinical care [11–14]. In the current intervention, we aimed to integrate the support steps in the working routines of HCPs and target their attitude, self-efficacy, and social influence. First, it is essential to be aware of the patient’s working status. After this, undiminished continuous encouragement of sustainable work participation is key in patient contact. In case of problems (or risk of them) revealed by screening, additional work-related support can be initiated, based on risk stratification, clinical reasoning, knowledge and skills of HCPs who feel supported by their colleagues (Fig. 1).

Whereas previous studies focused mainly on patients with an employer [12, 13], this intervention enables HCPs via behavioral change and the developed tools to provide support to all working patients, including self-employed patients or patients with flexible contracts.

It is important to emphasize that this work-related support is complementary and even synergistic when there is appropriate communication and cooperation with the patient’s occupational physician, if the patient has one. Where desirable and with the patient’s consent, the HCP can contact the occupational physician to discuss the proposed support [19, 34].

While testing the intervention, the limited time available to provide work-related support in clinical care appeared challenging. Therefore, the practical tools were developed to facilitate HCPs with identifying the problem and finding possible options for work-related support.

## Methodological considerations

This study comprises several strengths and limitations.

### Strengths

In our intervention development, traditional steps of IM were combined with action research principles to test intervention parts in practice while developing them [31, 32]. This approach resulted in a comprehensive needs assessment, leading to an intervention that addresses change in practice. HCPs were consulted in relation to each step of IM and invited to reflect on the support for their patients. Combining the two methods provided valuable insights. Importantly, testing of parts of the intervention did not lead to major changes in the intervention, which can be regarded as an initial proof of the intervention’s feasibility. Testing primarily pointed at the need for additional practical tools to increase efficiency, to obtain knowledge rather than only skills training, and at the need to feel supported by colleagues. Facilitating a social norm in which work-related support is regarded as normal and part of standard care also proved important. By including this practical knowledge in the development, the intervention was improved and better integrated with clinical care. However, this approach is time consuming, and only HCPs with interest in the topic of work participation were involved in the development.

Additionally, IM allows users (HCPs) to collaboratively integrate the developed intervention to their own working routines. The intervention is thus to be expected to fit HCPs’ needs.

Furthermore, the development resulted in practical tools, including screening card, conversation cards, Map with Options for Work-related Support (MOWS), and a flyer. In addition to being tools for behavioral change, these tools can have added value to provide work-related support during the limited consultation time of HCPs.

We focused on the behavioral change of the HCP, but the patient perspective was not neglected; it was taken into account as recommended [46] by additional research on patient needs (unpublished thesis, 2021).

Lastly, the intervention is generic and applicable to various stay-at-work populations, allowing for including disease-specific elements. The intervention is potentially applicable with some modifications to RTW populations, but this was not tested in this study.

### Limitations

Inevitably, this study contains some limitations. First, the intervention described has not yet been evaluated. However, a process evaluation and cost impact evaluation are planned. Second, while the current intervention is built upon a generic framework, we did not explicitly address steps how to implement the framework in various care settings.

## Implications for research

To encourage further research on similar interventions (or their development), it is important to better understand the views of HCPs regarding work-related support. Previous research has been conducted among patients [47–49], but more studies are recommended on the specific needs and views of HCPs on work-related support. The current intervention should be evaluated in terms of effects; a budget-impact analysis is recommended as well as a process evaluation among patients and HCPs.

## Implications for practice

By developing an intervention for different patient groups, a generic intervention was developed. However, the intervention cannot be directly adopted for all patients. Care pathways can differ considerably by setting, disease, and patient group. This care pathway was based on the local context. The local context refers to the hospital’s local care pathways with varying roles of HCPs, varying availability of resources (e.g. time, HCPs to provide support, institutional training, and information materials for patients), and varying availability of supportive local policies and national legislation.

In this pathway, attention is drawn to the chain, including a focus on work and tailored support by designated HCPs, such as a nurse specialist and medical specialist in rehabilitation medicine. HCPs’ limited time proved to be an important factor in providing work-related support within the existing consultation time, as well as attending training sessions within the existing working hours. To implement this intervention in practice, it is advised to facilitate the time of HCPs and arrange financing (e.g., within current hospital’s financial flows).

To apply this intervention to other patient groups, the disease-specific factors, local context, (financial) resources, and the national legislation should be duly considered [34]. In addition, caution is advised for its application to RTW populations, as this group will require different needs for such an intervention. This intervention was developed to give an initial impetus to work-related support in clinical care and offers opportunities to provide matched and patient-centered care.

## Conclusions

Combining Intervention Mapping with action research principles resulted in a generic stay-at-work intervention to provide work-related support in clinical care. The need for knowledge, skills, and practical tools appeared important. HCPs’ attitude, self-efficacy, and social influence were targeted to change their behavior. The development resulted in a generic care pathway to screen and identify patients who require tailored support. Practical tools, training sessions, and intervision were developed. To apply this intervention to other patient groups or implement the intervention in another hospital, disease-specific factors, the local context, (financial) resources, and the national legislation should be duly considered.

## Electronic Supplementary Material

Below is the link to the electronic supplementary material.

Supplementary Material 1

## Data Availability

Not applicable.
