# Correction to: Work-related support in clinical care for patients with a chronic disease: development of an intervention

**DOI:** 10.1007/s10926-022-10053-8

**Published:** 2022-06-11

**Authors:** Maarten Butink, Desiree Dona, Annelies Boonen, Marlies Peters, Vera Baadjou, Theo Senden, Angelique de Rijk

**Affiliations:** 1 Department of Internal Medicine, Division of Rheumatology, Care and Public Health Research Institute (CAPHRI), Maastricht University Medical Centre+ (MUMC+), P. Debyelaan 25, 6229 HX Maastricht, The Netherlands; 2 Department of Social Medicine, Care and Public Health Research Institute (CAPHRI), Maastricht University, Duboisdomein 30, 6200 MD Maastricht, The Netherlands; 3 Department of Human Resources/Occupational Health Services, Radboud University Medical Centre, Geert Grooteplein Zuid 10, 6525 GA Nijmegen, The Netherlands; 4 Department of Rehabilitation Medicine, Care and Public Health Research Institute (CAPHRI), Adelante Rehabilitation Centre/Maastricht University, Universiteitssingel 40, 6200 MD Maastricht, The Netherlands

## Correction to: Journal of Occupational Rehabilitation 10.1007/s10926-022-10032-z

In the original article there are two of the authors have a wrong affiliation. Author Theo Senden has affiliation 3 (Department of Human Resources/Occupational Health Services, Radboud University Medical Centre, Geert Grooteplein Zuid 10, 6525 GA, Nijmegen, The Netherlands) and author Angelique de Rijk (last author) has affiliation 2 (this is the same as affiliation 6).

The updated authors and their affiliation details are given in this erratum.

The Original article has been corrected.

