# Supplementary material for: Work-related support in clinical care for patients with a chronic disease: development of an intervention
Source: J Occup Rehabil. 2022 May 20;32(4):705–17. doi: 10.1007/s10926-022-10032-z (PMC9668761; doi:10.1007/s10926-022-10032-z)
Supplement: Supplementary file 1 — Supplementary Material 1 [file 10926_2022_10032_MOESM1_ESM.docx]

**Online Resources (1 and 2)**

**Online Resource 1.** Glossary

| **Term** | **Definition in the context of the work-related support intervention** |
| --- | --- |
| Clinical reasoning | The purposeful use, combination, weighing, and application of knowledge, cognitions, reflections, clinical experience, best practices, and observations by HCPs, to achieve patient-centered, work-related support that may also deviate from protocols or guidelines where necessary. |
| Designated HCP | HCP who has an active role in providing tailored work-related support for low- or high-complexity problems. |
| Follow-up | Aftercare by HCP (designated) to evaluate the provided work-related support and accommodate the initiated support if necessary. |
| HCP | Healthcare professional who has an active role in the process of screening and stratification while providing work-related support. |
| Problem assessment | A work-related problem assessment by designated HCPs together with the patient, focusing on the domains: health, followed by work, support, and personal circumstances. The assessment leads to an exploration and prioritization of possible options for work-related support. |
| Risk stratification | Strategy by HCPs to decide what work-related support should be initiated, guided by clinical reasoning. |
| Screening | Strategy by HCPs to check participants for being at risk for long-term sickness absence. |
| Tailored work-related support | Work-related support that is in line with an individual patient’s situation and preferences. |
| Work-related support for high-complexity problems | Support provided by a designated HCP for work-related problems that are generally more complex in nature or require more comprehensive approaches. An example is presented in Table 4. |
| Work-related support for low-complexity problems | Support provided by a designated HCP for work-related problems that are generally less complex in nature or require less complex support options.  An example is presented in Table 4. |

**Online Resource 2.** Format and content description of training sessions on work-related support

| Training session 1: Screening and stratification | Training session 2: Problem assessment and tailored support | Intervision |
| --- | --- | --- |
| Participants: HCPs  Teachers: two project group members  Location: hospital or online  Duration: 45 minutes (excl. preparation)  Materials/tools: handbook, presentation slides, knowledge clips, screening card | Participants: designated HCPs to provide tailored support  Teachers: two project group members  Location: online  Duration: 2 hours (excl. preparation)  Materials/tools: handbook, presentation slides, knowledge clips, conversation cards, Map with Options for Work-related Support (MOWS) | Participants: designated HCPs to provide tailored support Teachers: one project group member and/or one invited expert  Location: online  Duration per session: 1-2 hours (excl. preparation)  Materials/tools: - |
| Content  - basics of work-related support  - basics of social security system  - roles and responsibilities for the support of patients  - screening and use of screening card  - communication techniques  - stratification and referral  - follow-up  - practical examples | Content  - extended information on work-related support  - extended information on social security system  - roles and responsibilities for the support of patients  - problem assessment and use of conversation cards  - communication techniques  - providing work-related support options and use of the Map with Options for Work-related Support (MOWS) | Content  - discuss topics, cases, or challenges brought in by HCPs  - collaborative problem analysis  - collaborative solution definition |
